# Developing Research and Education on Aging Among Asians Through International Networks

**DOI:** 10.1093/geroni/igaa057.2953

**Published:** 2020-12-16

**Authors:** Wenjun Li

**Affiliations:** University of Massachusetts Medical School, Worcester, Massachusetts, United States

## Abstract

Asians are the largest and the fastest growing segment of the world population. However, age-related social and health issues are understudied among them. Studies on older Asians are often small-sized and scattered geographically, and use instruments that are inconsistent in context, language and culture. Findings from different studies on Asians are difficult to synthesize to support health promotion, clinical practice and policy evaluations. To advance research into aging among Asians, a fundamental step is to create content-relevant, linguistically and culturally appropriate research instruments, and encourage use of these consistent and comparable instruments across studies. In this interactive session, the author will lead the group to discuss the need, feasibility and strategies for developing an international network to promote resource sharing and research collaborations across country borders and disciplines. By bringing the isolated but passionate Asian aging researchers together, this network will accelerate the growth of research on Aging Among Asians.

